# Age-differential sexual dimorphism in CHD8-S62X-mutant mouse behaviors

**DOI:** 10.3389/fnmol.2022.1022306

**Published:** 2022-10-25

**Authors:** Soo Yeon Lee, Hanseul Kweon, Hyojin Kang, Eunjoon Kim

**Affiliations:** ^1^Department of Biological Sciences, Korea Advanced Institute for Science and Technology (KAIST), Daejeon, South Korea; ^2^Center for Synaptic Brain Dysfunctions, Institute for Basic Science (IBS), Daejeon, South Korea; ^3^Division of National Supercomputing, Korea Institute of Science and Technology Information, Daejeon, South Korea

**Keywords:** autism spectrum disorders, CHD8, mouse model of ASD, chromatin remodeling, sexual dimorphism, age dependence

## Abstract

Autism spectrum disorders (ASD) are ~4-times more common in males than females, and CHD8 (a chromatin remodeler)-related ASD shows a strong male bias (~4:1), although the underlying mechanism remains unclear. *Chd8*-mutant mice with a C-terminal protein-truncating mutation (N2373K) display male-preponderant behavioral deficits as juveniles and adults, although whether this also applies to other *Chd8* mutations remains unknown. In addition, it remains unclear whether sexually dimorphic phenotypes in *Chd8*-mutant mice are differentially observed in males and females across different ages. We here generated new *Chd8*-mutant (knock-in) mice carrying a patient-derived mutation causing an N-terminal and stronger protein truncation (*Chd8^+/S62X^* mice) and characterized the mice by behavioral analyses. Juvenile *Chd8^+/S62X^* mice displayed male-preponderant autistic-like behaviors; hypoactivity and enhanced mother-seeking/attachment behavior in males but not in females. Adult male and female *Chd8^+/S62X^* mice showed largely similar deficits in repetitive and anxiety-like behavioral domains. Therefore, the CHD8-S62X mutation induces ASD-like behaviors in juvenile male mice and adult male and female mice, pointing to an age-differential sexual dimorphism and also distinct sexual dimorphisms in different *Chd8* mutations (N2373K and S62X).

## Introduction

Autism spectrum disorders (ASD) are ~4-times more common in males than in females (Werling and Geschwind, 2013). This male–female difference depends on which ASD-risk genes are affected, with genes including *CHD8*, *ANK2*, *ADNP*, and *TRIP12* showing a strong male bias (Stessman et al., 2017; Andreae and Basson, 2018). However, the underlying mechanisms remain unclear.

CHD8, a chromatin remodeler, is strongly associated with ASD, accounting for ~0.5% cases (Bernier et al., 2014; Barnard et al., 2015). Among ASD patients carrying *CHD8* mutations, the male–female ratio is high (~85:15) (Stessman et al., 2017). Previous studies on *Chd8*-mutant mice have revealed various mechanisms underlying CHD8-related brain dysfunctions in ASD (Sugathan et al., 2014; Cotney et al., 2015; Wang et al., 2015; Breuss and Gleeson, 2016; Durak et al., 2016; Katayama et al., 2016; Gompers et al., 2017; Platt et al., 2017; Andreae and Basson, 2018; Jung et al., 2018; Suetterlin et al., 2018; Wade et al., 2018; Xu et al., 2018; Zhao et al., 2018; Hulbert et al., 2020; Jimenez et al., 2020; Sood et al., 2020; Cherepanov et al., 2021; Ellingford et al., 2021; Hurley et al., 2021; Kawamura et al., 2021; Kweon et al., 2021; Weissberg and Elliott, 2021; Yu et al., 2022).

Our previous study reported that *Chd8*-mutant (knock-in) mice with a patient-derived, C-terminal protein-truncating mutation (N2373K) display male-preponderant behavioral deficits at pup, juvenile, and adult stages that are associated with altered neuronal, synaptic, and transcriptomic phenotypes (Andreae and Basson, 2018; Jung et al., 2018). In addition, female mice with the mutation display unique transcriptomic and synaptic/neuronal changes that are different from those in males. However, whether other *CHD8* mutations induce similar sexually dimorphic phenotypes in mice remains largely unclear (Cherepanov et al., 2021; Yu et al., 2022), partly because male mice were mainly used for phenotypic analyses, or males and female mice were not separated in experimental mouse cohorts (Gompers et al., 2017; Suetterlin et al., 2018; Hulbert et al., 2020; Jimenez et al., 2020; Ellingford et al., 2021; Hurley et al., 2021; Kawamura et al., 2021).

In the present study, we generated new *Chd8*-mutant (knock-in) mice carrying a patient-derived mutation causing an N-terminal and stronger protein truncation (CHD8-S62X). Adult *Chd8^+/S62X^* mice displayed behavioral deficits that are largely similar, but such deficits were more strongly observed in juvenile males but not females. These results suggest an age-dependent sexual dimorphism in *Chd8^+/S62X^* mice.

## Materials and methods

### Animals

C57BL/6 J mice with the S62X knock-in mutation in the *Chd8* gene, flanked by loxP sites and a neomycin cassette, were designed and generated by Cyagen. The neomycin cassette was removed by crossing heterozygous (HT) mice with protamine-Flp mice. WT and HT mice were genotyped by PCR using the following primers: 5’-GTC AGC TAG CTC AGG CTG CT-3′ (forward), 5′-GGT ACA GCG CAG CAT GTG AT-3′ (reverse). Mice were maintained at the mouse facility of the Korea Advanced Institute of Science and Technology (KAIST) and were housed under a 12-h light–dark cycle with unlimited access to food and water.

### Brain weight and size

Brains were dissected, placed in mouse brain matrices, and coronally cut below the cortex. After measuring the weight, brains were imaged by Gel Doc. A-P (anterior–posterior) length, cortical length, and cortical area were analyzed by ImageJ.

### Immunohistochemistry

Mice were cardiac perfused with 4% paraformaldehyde (PFA), and brains were dissected. After storing the dissected brains in 4% PFA for more than 1 day, coronal sections (50 μm) were prepared using a vibratome (Leica). Brain slices were blocked with 5% normal donkey serum and 0.2% TritonX-100 for 1 h and incubated with primary antibodies (1:500 NeuN) overnight. After washing, slices were incubated with fluorophore-conjugated secondary antibodies (1,1,000) in phosphate-buffered saline with 0.2% Triton X-100. After washing, sections were mounted with Vectorshield (Vector Laboratory), and images were acquired using a slide scanner (Axio Scan Z1, Zeiss).

### Western blot

Protein lysates from the whole brain were prepared and loaded into gels that were manually made. Proteins were transferred to the PVDF membrane, blocked with 5% skim milk, and stained overnight using CHD8 (Bethyl, A301-224A) and β-actin (Sigma A5316) antibodies. Membranes were washed and stained with HRP-conjugated secondary antibodies. Signals were quantified using Image Studio Lite.

### Behavioral assays

Mice at P19–28 were used for juvenile behavioral experiments. For adult experiments, mice at the age of 9–16 weeks were used. Before the testing day, the mice were handled for 10 min for 3 days to minimize stress from human handling. Mice were placed in a dark test room for 30 min for habituation just before the behavioral experiments. All behavioral assays were performed during the light-off period when mice are active unless otherwise specified. All data were analyzed using Ethovision XT (Noldus) by an experimenter blind to the genotype unless otherwise specified. Behavioral assays were carried out in the order of increasing stress to minimize the effects of stress. Details of these assays are explained below in the order they were carried out.

### Open-field test

Mice were placed in an open field box (40 × 40 × 40 cm), and the behaviors were recorded for 60 min for adult mice or 20 min for juvenile mice. The intensity of light in the box was set at 100 lux for adult mice and 15 lux for juvenile mice. The distance moved and time spent in the center region of the open-field box (20 × 20 cm) were measured as a measure of anxiety-like behavior.

### Repetitive behaviors

To measure the duration of self-grooming and digging, representing repetitive behaviors, mice were placed in a new home cage with fresh bedding, and their behaviors were recorded for 30 min. Mice spent much more time digging than self-grooming. To measure the duration of self-grooming without being confounded by digging behavior, mice were placed in a specially designed chamber (15 × 15 × 40 cm), and their behaviors were recorded for 10 min. Light intensity in the cage was set to 50 lux to minimize light-induced anxiety. The first 10 min was considered as a habituation period, and self-grooming and digging durations during the last 20 min of the recordings were quantified manually.

### Light/dark test

Mice were placed in a box that contains a white open box conjoined to a closed black (dark) box. There was a small entrance to allow the mice to move freely between these two boxes. The light intensity in the light box was set to 300 lux to examine light-induced anxiety. Behaviors were recorded for 20 min, and the time spent in each box was measured.

### Elevated plus-maze test

Mice were placed in the elevated plus-maze with two open arms and two closed arms (5 × 30 cm each, with 30 cm-tall walls in the closed arm). The maze was placed 50 cm above the ground. The intensity of light in the center region connected to all four arms was set to 200 lux. Behaviors were recorded for 10 min. Times spent in closed and open arms and the total distance moved were measured.

### Three-chamber test

For the three-chamber (Silverman et al., 2010) test, mice were isolated for 3 days before the experiment to avoid the effect of prior interactions. A mouse was placed in an empty three-chamber apparatus (40 × 60 cm) and allowed to explore the environment and habituate for 10 min. Light intensity was set to 50 lux. Following habituation, a small cage containing a stranger mouse (129/SvJae strain; S1) was placed in one of the corners. Another cage containing an object (O) was placed in the other corner of the apparatus, followed by recordings for 10 min. After 10 min, the object was replaced with another stranger mouse (S2), and the behaviors were recorded for 10 min. Time spent sniffing targets (object/stranger) was measured.

### Dyadic social interaction

After 3-day social isolation, mice were habituated in the open-field box for 10 min a day before testing. On the test day, two mice with identical genotypes and sex that have not met each other before were placed in the open-field box, and their behaviors were recorded for 10 min. The duration of social interactions was quantified manually. Light intensity was set to 50 lux.

### Juvenile play

Mice were isolated and habituated in their home cage with fresh bedding for 10 min a day before testing. On the test day, two mice with identical genotypes and sex that have not met each other before were placed in a new home cage with fresh bedding, and their behaviors were recorded for 10 min. Light intensity was set to 15 lux. The duration of social interactions was quantified manually.

### Juvenile maternal homing

The juvenile maternal homing test was performed as previously described (Zhan et al., 2014). Mice at P19 were separated from their mothers at least 30 min before the test. The test consists of two phases; a nest-homing phase followed by a maternal homing phase. In the nest-homing phase, bedding materials from the home cage where the separated mouse used to spend time with the mother and siblings (Nest) and fresh bedding (New) were placed in the opposite corners of an open field box (40 × 40 × 40 cm). Mice were placed in one of the empty corners, and behaviors were recorded for 3 min. In the maternal homing phase, an empty chamber and a chamber containing the mother of the subject mouse were placed in the two remaining empty corners of the box. Mice were placed in the corner with home-cage beddings (Nest), and their behaviors were recorded for 5 min. Light intensity in the center of the box was set to 15 lux, and time spent at each corner was measured.

### LABORAS analysis

The Laboratory Animal Behavior Observation Registration and Analysis System (LABORAS, Metris) is a system that allows the quantification of mouse behaviors over a long period of time (usually days) without perturbation by researchers (Quinn et al., 2003). The system consists of a cage placed above a vibration-sensitive platform and a connected program (LABORAS 2.6). Mice were individually placed in the apparatus, and their behaviors were recorded for 96 h. The first 24 h were considered as the time for habituation and were not analyzed. Data analysis was performed using LABORAS 2.6 program (Metris).

### Ultrasonic vocalization

Male mice were placed in a home cage with an age-matched unfamiliar C57BL/6 J female mouse. For pup USV, pups were separated from their mothers and placed in a plastic container. Ultrasonic vocalizations (USVs) were recorded for 5 min using an ultrasound microphone (Avisoft) and Avisoft Recorder software. Recorded USVs were analyzed as previously described (Kim et al., 2018).

### Analysis and statistics

All experiments were performed and analyzed by researchers blind to the genotype. Outliers were identified and excluded using ROUT test (Q = 1%). To compare male and female mouse phenotypes, two-way ANOVA with sex and genotype as main variables was used. For two-way ANOVA, normality was assumed. Statistical tests were performed using Graphpad Prism 9 and SigmaPlot 12.0. Statistical details, including the sex, age, and number of mice, are described in Supplementary Table 1.

## Results

### Generation and basic characterization of male and female *Chd8^+/S62X^* mice

**Figure 1:**
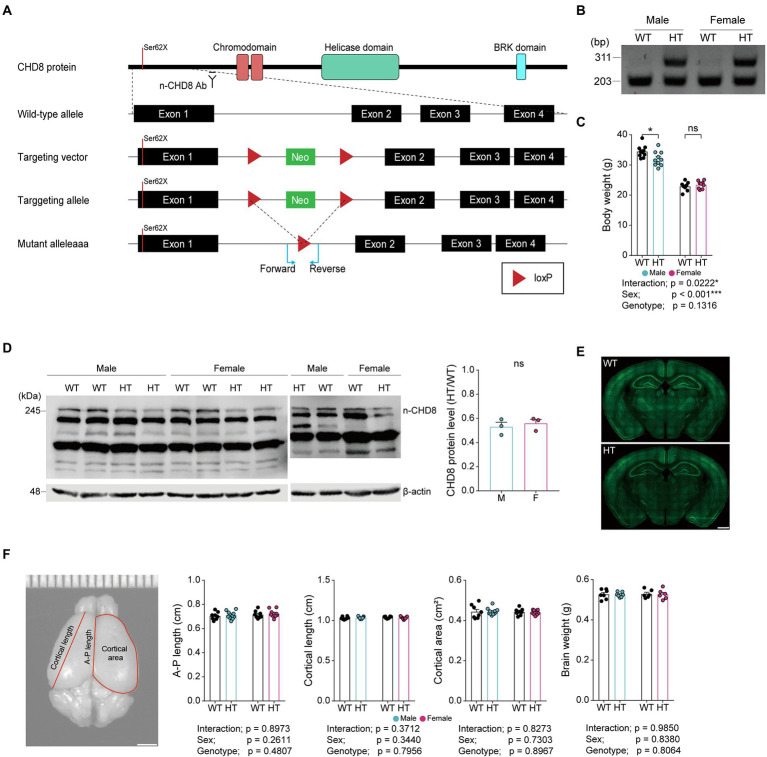
CHD8-S62X knock-in strategy, PCR genotyping, body weight, CHD8 protein levels, gross brain morphology, and brain size in Chd8^+/S62X^ male and female mice. **(A)** Chd8 knock-in (S62X) strategy. **(B)** PCR genotyping of WT and Chd8^+/S62X^ mice. WT, wild-type; HT, heterozygote (*Chd8^+/S62X^*). **(C)** *Chd8^+/S62X^* male and female mice (4 months) show largely normal postnatal body weights compared with WT mice. (n = 10 mice [male-WT], 10 [male-HT/heterozygote], 8 [female-WT], and 9 [female-HT], *p < 0.05, ns, not significant, two-way ANOVA with Holm-Sidak test). **(D)** *Chd8^+/S62X^* male and female mice (P15) show the expected ~50% decrease in total brain levels of the CHD8 protein compared with WT mice, as shown by immunoblotting of whole-brain lysates. (*n* = 3 mice [male-WT], 3 [male-HT], 3 [female-WT], and 3 [female-HT], ns, not significant, Student’s *t*-test). Other bands indicate non-specific protein bands that cross-react with the antibody. **(E)** *Chd8^+/S62X^* male and female mice (4 months) show largely normal gross brain morphology, as shown by NeuN (neuronal marker) staining. **(F)** *Chd8^+/S62X^* male and female mice (4 months) show largely normal brain sizes compared with WT mice, as shown by anterior–posterior (A–P) length, cortical length, and cortical area of the brain. (*n* = 8 mice [male-WT], 11 [male-HT], 8 [female-WT], and 9 [female-HT], two-way ANOVA).

To test if a heterozygous (HT) CHD8-S62X mutation differentially induces autistic-like behaviors in male and female mice, we generated and characterized *Chd8^+/S62X^* mice, carrying a patient-derived N-terminal protein-truncating mutation (Figures 1A,B; Supplementary Figure 1; OʼRoak et al., 2012). Male and female *Chd8^+/S62X^* mice, with CHD8 protein levels ~50% of wild-type (WT) mice, were born at normal Mendelian ratios and exhibited largely normal body weight, gross brain morphology, and brain size (Figures 1C–F).

### Hypoactivity and enhanced mother-seeking/attachment behavior in male but not female *Chd8^+/S62X^* juveniles

Given that ASD is characterized by early symptomatic manifestations and *Chd8^+/N2373K^* mice (C-terminal truncation) showed autistic-like enhanced anxiety-like behaviors in pup and juvenile stages (Jung et al., 2018), we examined early behavioral deficits in *Chd8^+/S62X^* mice.

**Figure 2:**
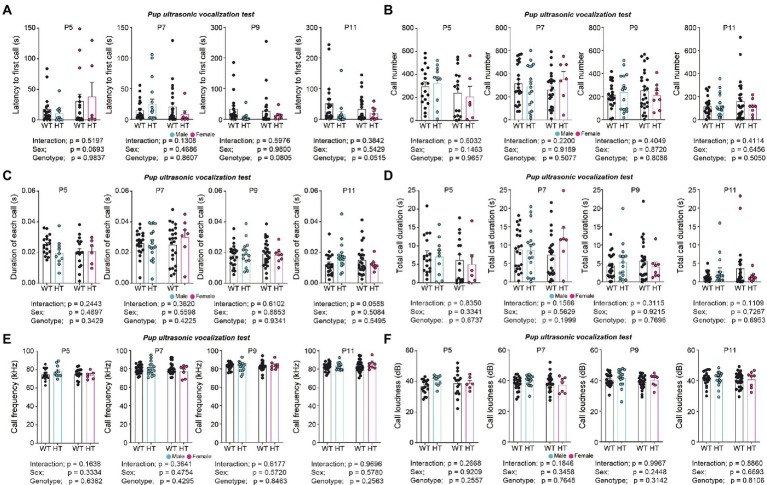
Largely normal USVs in pups separated from their mothers in *Chd8^+/S62X^* male and female mice. **(A)** Latency to first call in WT and *Chd8^+/S62X^* pups at P5, P7, P9, and P11. WT, wild-type; HT, heterozygote (*Chd8^+/S62X^*). (*n* = 21 mice [male-WT], 17 [male-HT/heterozygote], 26 [female-WT], and 8 [female-HT], ns, not significant, two-way ANOVA). **(B)** Number of USV calls in WT and *Chd8^+/S62X^* pups. (*n* = 21 mice [male-WT], 17 [male-HT/heterozygote], 26 [female-WT], and 8 [female-HT], ns, not significant, two-way ANOVA). **(C)** Duration of each call in WT and *Chd8^+/S62X^* pups. (*n* = 21 mice [male-WT], 17 [male-HT/heterozygote], 26 [female-WT], and 8 [female-HT], ns, not significant, two-way ANOVA). **(D)** Total duration of USV calls in WT and *Chd8^+/S62X^* pups. (*n* = 21 mice [male-WT], 17 [male-HT/heterozygote], 26 [female-WT], and 8 [female-HT], ns, not significant, two-way ANOVA). **(E)** USV call frequency in WT and *Chd8^+/S62X^* pups. (*n* = 21 mice [male-WT], 17 [male-HT/heterozygote], 26 [female-WT], and 8 [female-HT], ns, not significant, two-way ANOVA). **(F)** USV call amplitude in WT and *Chd8^+/S62X^* pups. (*n* = 21 mice [male-WT], 17 [male-HT/heterozygote], 26 [female-WT], and 8 [female-HT], ns, not significant, two-way ANOVA).

*Chd8^+/S62X^* pups separated from their mothers emitted largely normal levels of USVs, except for a tendency for a moderate decrease in the latency to first call, compared with WT mice (Figures 2A–F; see Supplementary Table 1 for statistical details).

**Figure 3:**
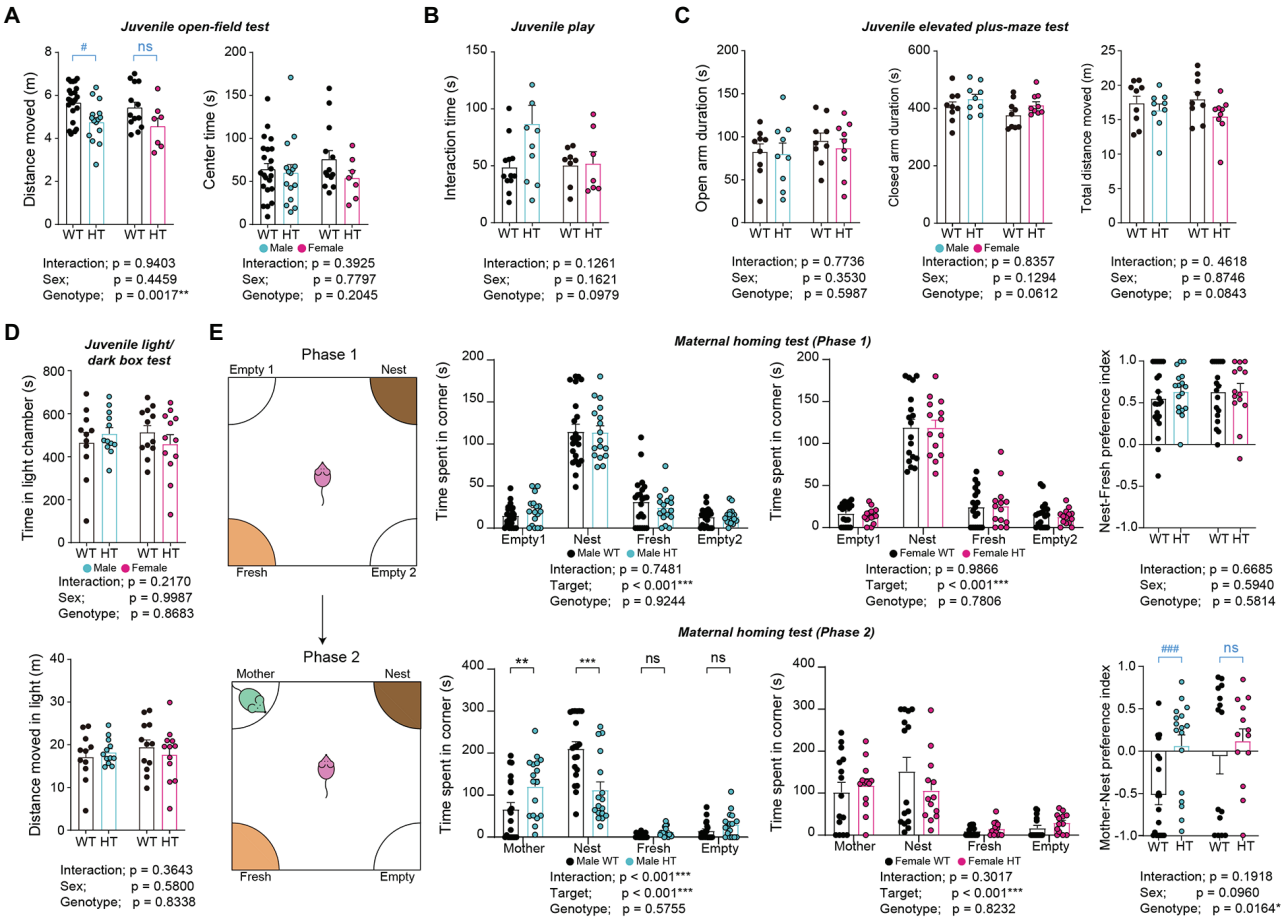
Hypoactivity and enhanced mother-seeking/attachment behavior in male but not female *Chd8^+/S62X^* juveniles. **(A)** Juvenile male and female *Chd8^+/S62X^* mice (P24) show genotype-dependent decreases in the locomotor activity in the open-field test compared with WT mice, although the center-zone activity is normal, as shown by the total distance moved and time spent in the center region of the open-field arena and supported by two-way ANOVA. Note, however, that male, but not female, *Chd8^+/S62X^* juveniles show hypoactivity, in the Mann–Whitney U test, which was performed in addition to two-way ANOVA for the lack of significant genotype-sex interaction; the Mann–Whitney U test results are indicated in blue labels above the bar graphs. (*n* = 22 mice [male-WT], 15 [male-HT], 13 [female-WT], and 7 [female-HT], **p < 0.001, two-way ANOVA, ^#^p < 0.05, ns, not significant, Mann–Whitney *U* test). **(B)** Juvenile male and female *Chd8^+/S62X^* mice (P22) show largely normal direct social interaction in the juvenile play test, compared with WT mice, as shown by total interaction time (nose-to-nose, nose-to-tail, following, mounting, and allo-grooming). (*n* = 12 mouse pairs [male-WT], 11 [male-HT], 8 [female-WT], and 7 [female-HT], two-way ANOVA). **(C)** Juvenile male and female *Chd8^+/S62X^* mice (P26) spend normal amounts of time in closed and open arms in the elevated plus-maze test compared with WT mice. (*n* = 9 [male-WT], 9 [male-HT], 9 [female-WT], and 9 [female-HT], two-way ANOVA). **(D)** Juvenile male and female *Chd8^+/S62X^* mice (P28) spend normal amounts of time in the light chamber of the light–dark apparatus compared with WT mice. (n = 11 mice [male-WT], 12 [male-HT], 12 [female-WT], and 12 [female-HT], two-way ANOVA). **(E)** Male juvenile *Chd8^+/S62X^* mice (P19) show enhanced mother-seeking/attachment behavior, whereas female juvenile *Chd8^+/S62X^* mice do not. In the first phase of the maternal-homing test, male and female juvenile *Chd8^+/S62X^* mice separated from their mothers in the maternal homing test spend comparable amounts of time sniffing familiar home-cage nest, or fresh, material, as compared with WT mice. In the second phase, male, but not female, *Chd8^+/S62X^* mice spend more time with the reunited mother and less time with the home-cage nest material, as compared with WT mice. (n = 22 mice [male-WT], 17 [male-HT], 18 [female-WT], and 14 [female-HT] for phase 1, and 20 [male-WT], 17 [male-HT], 15 [female-WT], and 13 [female-HT] for phase 2,**p < 0.01, ***p < 0.001, ns, not significant, two-way ANOVA with Holms-Sidak test [separate ANOVA for males or females], *p < 0.05, two-way ANOVA [preference index], ^###^*p* < 0.001, ns, not significant, Mann–Whitney *U* test [within-sex comparisons of preference index]).

Juvenile male and female *Chd8^+/S62X^* mice showed decreased levels of locomotor activity in the open-field test compared with WT mice, although center-zone activity was normal, as supported by a significant genotype difference (Figure 3A). However, the Mann–Whitney U test performed for the lack of the genotype-sex interaction, which does not allow multiple comparisons, revealed hypoactivity in males but not in females, although there was a tendency for hypoactivity in females. Male and female *Chd8^+/S62X^* mice also showed normal levels of anxiety-like behaviors in elevated plus-maze and light–dark tests and social interaction in the juvenile play test, compared with WT mice (Figures 3B–D).

Intriguingly, in the maternal homing test, in which a juvenile mouse is separated from its mother for 30 min and given the nest material from the home cage that it shared with its mother, male and female *Chd8^+/S62X^* mice spent similar amounts of time with the home-cage, or fresh, nest materials, compared with WT mice, as shown by time spent in targets (i.e., home-cage/nest and fresh) and the nest-fresh preference index (difference in time spent for different targets over total time spent) (Figure 3E).

In the second phase of the test, in which juvenile mice are allowed to reunite with their mother, male, but not female, *Chd8^+/S62X^* mice spent more time with the reunited mother and less time with the home-cage nest material, compared with WT mice, as shown by time spent in targets (i.e., mother and nest) and the mother-nest preference index (Figure 3E). The Mann–Whitney U test was also used here for the lack of the genotype-sex interaction.

These results suggest largely normal social communication in *Chd8^+/S62X^* pups and largely normal anxiety-like behavior in juvenile *Chd8^+/S62X^* males and females but hypoactivity and anxiety-like mother-seeking/attachment behaviors in male *Chd8^+/S62X^* juveniles but not in female *Chd8^+/S62X^* juveniles.

### Largely normal social behaviors in adult male and female *Chd8^+/S62X^* mice

**Figure 4:**
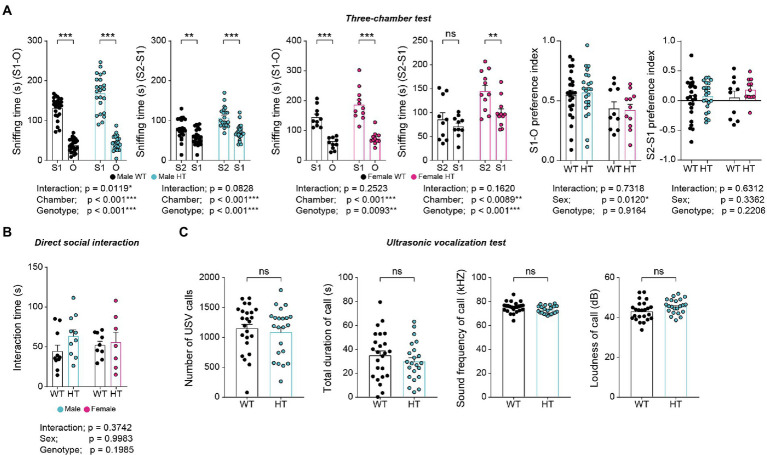
Normal social behaviors in adult male and female *Chd8^+/S62X^* mice. **(A)** Male and female *Chd8^+/S62X^* mice (3  months) show normal levels of social approach and social novelty recognition in the three-chamber test compared with WT mice, as shown by time spent sniffing a social stranger (S1) versus object (O), or a novel stranger (S2) versus a familiar stranger (S1), and the preference index (difference in time spent sniffing different targets over total time spent sniffing). (*n* = 24 mice [male-WT], 23 [male-HT/heterozygote], 10 [female-WT], and 11 [female-HT], *p < 0.05, **p < 0.01, ***p < 0.001, ns, not significant, two-way ANOVA with Holm-Sidak test [separate ANOVA for males or females], *p < 0.05, two-way ANOVA [preference index]). **(B)** Male and female *Chd8^+/S62X^* mice (3  months) show normal levels of direct social interaction in the dyadic social-interaction test compared with WT mice, as shown by total interaction time (nose-to-nose, nose-to-tail, following, mounting, and allo-grooming). (*n* = 10 mouse pairs [male-WT], 10 [male-HT], 9 [female-WT], and 7 [female-HT], two-way ANOVA). **(C)** Male *Chd8^+/S62X^* mice (3–4  months) show normal levels of USVs upon encountering a female stranger compared with WT mice, as shown by the number of calls, duration of each call, call sound frequency, and call loudness. (*n* = 24 mice [WT], 22 [HT], ns, not significant, Student’s *t*-test).

Adult male and female *Chd8^+/S62X^* mice showed largely normal social approach and social novelty recognition in the three-chamber test compared with WT mice, as supported by time spent sniffing targets and the preference index (difference in time spent for different targets over total time spent) (Figure 4A). Males and females also showed normal levels of direct social interaction in the dyadic social-interaction test compared with WT mice (Figure 4B).

Upon encountering a novel female mouse, male *Chd8^+/S62X^* mice emitted normal levels of courtship ultrasonic vocalizations (USVs) compared with WT mice (Figure 4C). These results suggest largely normal social behaviors and communication in adult male and female *Chd8^+/S62X^* mice.

### Differential increases in repetitive behaviors in adult male and female *Chd8^+/S62X^* mice

**Figure 5:**
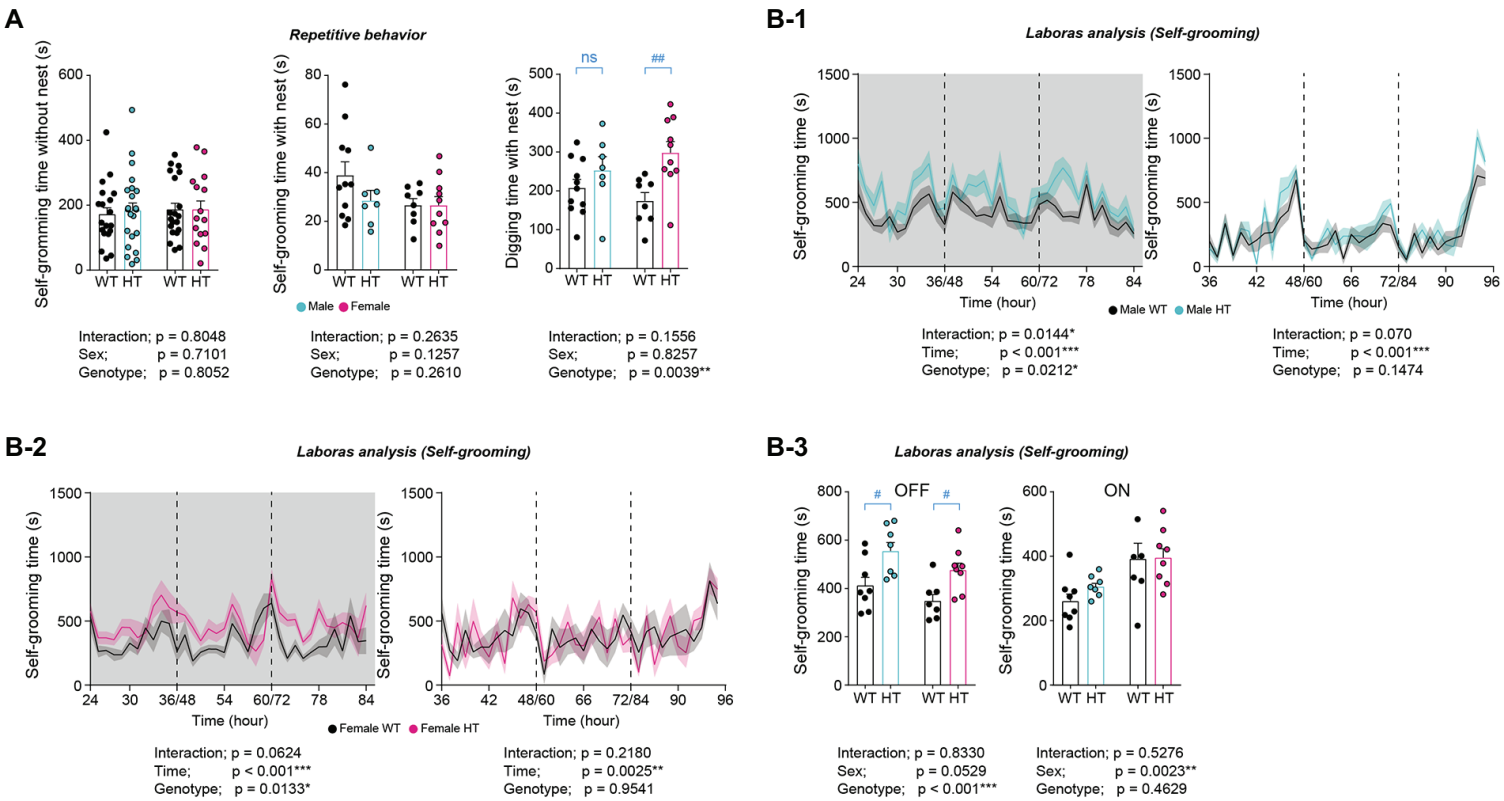
Differential increases in repetitive behaviors in adult male and female *Chd8^+/S62X^* mice. **(A)** Normal self-grooming but altered digging behaviors in male and female *Chd8^+/S62X^* mice (3–4  months) in novel home cages compared with WT mice. (*n* = 11 mice [male-WT], 7 [male-HT], 8 [female-WT], and 10 [female-HT], **p < 0.01, two-way ANOVA, ^##^*p* < 0.01, ns, not significant, Mann–Whitney *U* test and Student’s *t*-test). **(B)** Similarly increased self-grooming in LABORAS cages (a familiar environment) in adult male and female *Chd8^+/S62X^* mice (2  months), compared with WT mice. Light-off and light-on phases, which were pooled from the three-day recordings, are indicated in grey and white backgrounds, respectively. (*n* = 8 mice [male-WT], 7 [male-HT], 7 [female-WT], and 8 [female-HT], *p < 0.05, **p < 0.01, ***p < 0.001, two-way repeated-measures/RM-ANOVA with Holm-Sidak test [B-1-B-3], ^#^*p* < 0.05, Mann–Whitney *U* test [B-3]).

In tests of repetitive behaviors, adult male and female *Chd8^+/S62X^* mice showed similarly increased digging but normal self-grooming behavior in novel home cages, as supported by two-way ANOVA (significant genotype difference for digging) (Figure 5A). However, additional Mann–Whitney U test and t-test for digging within males or females indicated an increase in digging in females but not in males.

In LABORAS cages, a familiar environment where mouse movements are monitored for consecutive 72 h, male and female *Chd8^+/S62X^* mice showed similarly increased self-grooming in light-off phases, as supported by two-way ANOVA and the Mann–Whitney U test (Figure 5B). These results collectively that adult male and female *Chd8^+/S62X^* mice show differential increases in repetitive behaviors.

### Differential increases in anxiety-like behaviors in adult male and female *Chd8^+/S62X^* mice

**Figure 6:**
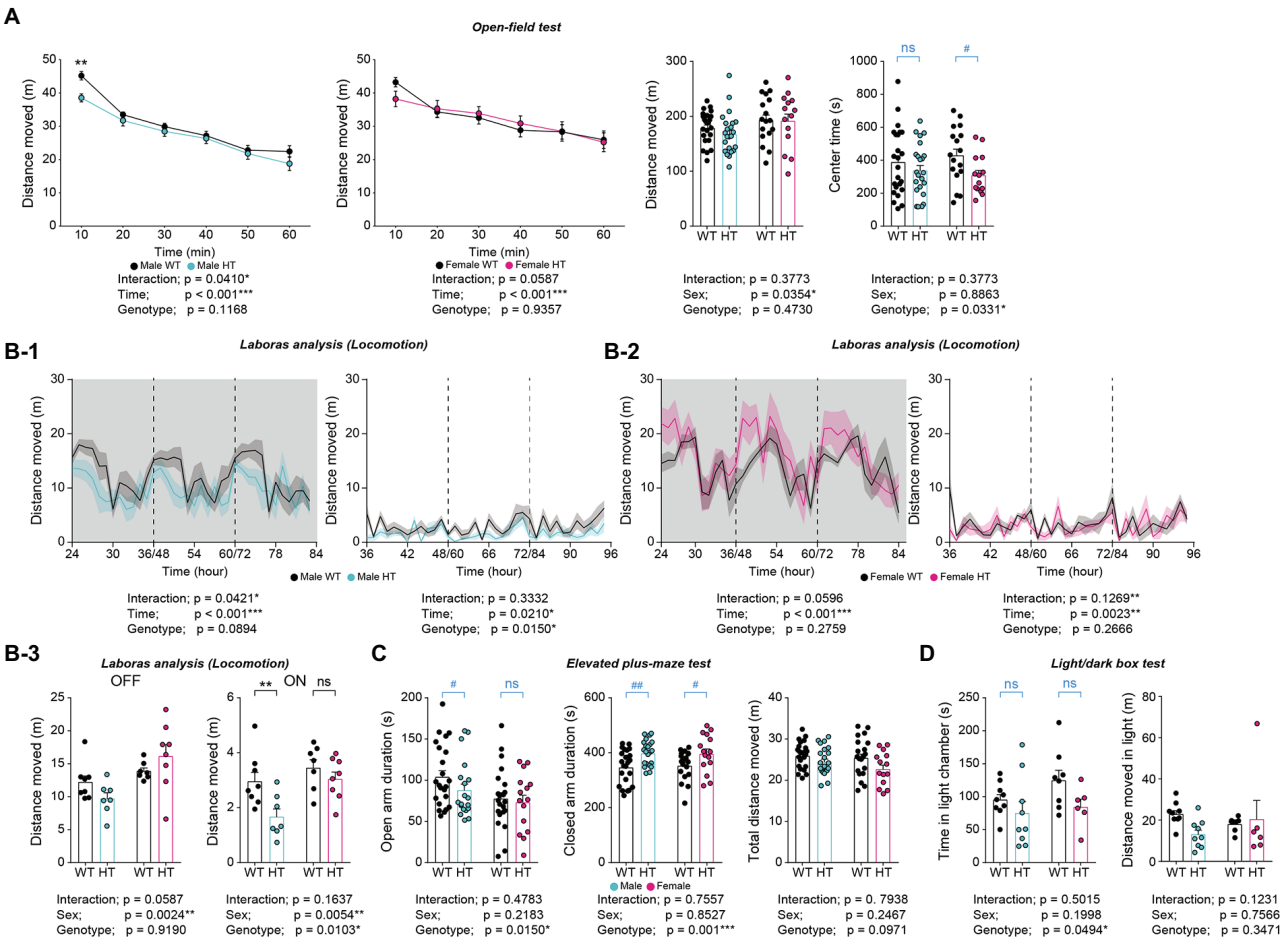
Differential increases in anxiety-like behaviors in adult male and female *Chd8^+/S62X^* mice. **(A)** Male and female *Chd8^+/S62X^* mice (2–3 months) show normal levels of locomotor activity in the open-field test, compared with WT mice, as shown by the distance moved and supported by two-way ANOVA. Note, however, that there is a genotype difference (decrease) in the center time in *Chd8^+/S62X^* mice, suggestive of anxiety-like behavior, although additional *t*-tests, performed for the lack of genotype-sex interaction, reveal a significant decrease in females but not males. (*n* = 24 mice [male-WT], 23 [male-HT], 17 [female-WT], and 14 [female-HT], *p < 0.05, ***p < 0.001, two-way RM-ANOVA, *p < 0.05, two-way ANOVA, ^#^*p* < 0.05, ns, not significant, Student’s *t*-test [within sex]). **(B)** Male and female *Chd8^+/S62X^* mice (2  months) show largely normal levels of locomotor activity in LABORAS cages compared with WT mice, as shown by total distance moved during light-off and light-on phases. Note that light-on, but not light-off, locomotor activity is decreased in males but not in females, (*n* = 8 [male-WT], 7 [male-HT], 7 [female-WT], and 8 [female-HT], *p < 0.05, **p < 0.01, ***p < 0.001, ns, not significant, two-way RM-ANOVA), *p < 0.05, **p < 0.01, ns, not significant, two-way ANOVA with Holm-Sidak test). **(C)** Time spent in open/closed arms in the elevated plus-maze test in male and female *Chd8^+/S62X^* mice (2–3 months) compared with WT mice. Note that male mutant mice show decreased open-arm time and increased closed-arm time, compared with WT mice, while female mutant mice show only increased closed-arm time. (*n* = 23 [male-WT], 19 [male-HT], 22 [female-WT], and 15 [female-HT], *p < 0.05, ***p < 0.001, two-way ANOVA, ^#^*p* < 0.05, ^##^p < 0.01, ns, not significant, Student’s *t*-test and Mann–Whitney *U* test). **(D)** Male and female *Chd8^+/S62X^* mice (3–4 months) spend less time in the light chamber of the light–dark apparatus compared with WT mice. (*n* = 9 mouse pairs [male-WT], 9 [male-HT], 8 [female-WT], and 6 [female-HT], *p < 0.05, two-way ANOVA, ns, not significant, Student’s *t*-test and Mann–Whitney *U* test).

In the open-field test, adult male and female *Chd8^+/S62X^* mice showed largely normal locomotor activity (i.e., comparable to that of WT mice) in both novel home cages and familiar LABORAS cages (Figures 6A,B).

However, *Chd8^+/S62X^* mice spent less time in the center region of the open-field area compared with WT mice, suggestive of anxiety-like behavior, as supported by a significant genotype difference (Figure 6A). However, additional t-tests performed for the lack of the genotype-sex interaction revealed a decrease in center-region time in mutant females but not in mutant males.

In addition, *Chd8^+/S62X^* mice spent less time in open arms of the elevated plus-maze and more time in the closed arms, as supported by the genotype difference (Figure 6C), further suggesting enhanced anxiety-like behaviors in adult *Chd8^+/S62X^* mice. However, additional t-test and Mann–Whitney U test indicated that male *Chd8^+/S62X^* mice showed decreased open-arm time and increased closed-arm time while female *Chd8^+/S62X^* mice showed only increased closed-arm time.

In the light–dark test, *Chd8^+/S62X^* mice spent less time in the light chamber compared with WT mice, although additional t-test and Mann–Whitney U test indicated insignificant changes in both male and female mutant mice compared with WT mice (Figure 6D). Therefore, adult male and female *Chd8^+/S62X^* mice display differential increases in anxiety-like behaviors.

## Discussion

In the present study, we investigated the impacts of a patient-derived, N-terminal protein-truncating CHD8 mutation (S62X) on male and female mice at behavioral levels across postnatal stages. Our results indicate that the CHD8-S62X mutation induces age-differential sexual dimorphism in behavioral deficits.

The current results indicate that repetitive and anxiety-like behavioral deficits are observed in adult male and female *Chd8^+/S62X^* mice, whereas hypoactivity and excessive mother seeking/attachment are mainly observed in juvenile *Chd8^+/S62X^* males. Statistical analyses of the results by two-way ANOVA frequently yielded significant genotype differences but the lack of genotype-sex interactions, pointing to similar directions in the behavioral changes in male and female *Chd8^+/S62X^* mice at juvenile and adult stages. Additional t-tests, however, indicated an interesting difference between *Chd8^+/S62X^* juveniles and adults; juvenile males but females showed behavioral deficits (hypoactivity and excessive mother seeking), while adult males and females showed largely similar deficits (repetitive behaviors and anxiety-like behaviors). Therefore, juvenile male-preponderant behavioral deficits seem to be changed to adult behavioral deficits that are similarly observed in males and females, suggestive of age-differential sexually dimorphic behavioral deficits in *Chd8^+/S62X^* mice. These results differ from the male-preponderant behavioral deficits that are persistently observed in *Chd8^+/N2373K^* mice across pup, juvenile, and adult stages (Jung et al., 2018).

The current results also suggest age-differential behavioral deficits in *Chd8^+/S62X^* mice. In the anxiety-like behavioral domain, *Chd8^+/S62X^* pups show largely normal mother separation-induced USVs, while *Chd8^+/S62X^* juveniles show partially increased anxiety-like behaviors (increased mother seeking but normal open-field center time [males]), which is followed by increased anxiety-like behavior in adults (decreased open-field center time [females] and increased closed-arm time [males and females]. In the locomotor activity domain, *Chd8^+/S62X^* juveniles (males) show open-field hypoactivity, whereas *Chd8^+/S62X^* adults (males and females) show normal open-field activity, further supporting the idea that deficits in a particular behavioral domain change across postnatal stages in *Chd8^+/S62X^* juveniles. These results collectively suggest that different *CHD8* mutations could lead to different patterns of male–female differences with varying severities and time courses of development. These data will help design future experiments with different research focuses such as male–female differences, temporal phenotypic alterations, and phenotypic strengths.

ASD exhibits the largest male–female ratio (~4–5:1) among neuropsychiatric disorders (Werling and Geschwind, 2013). Previous studies have shown that the male–female ratio can differ depending on the identity of the ASD-risk genes involved (Stessman et al., 2017). Our study extends this concept by showing that, even within the same gene, two different mutations (N2372K vs. S62X) can change the extent and time course of sexual dimorphisms in autistic-like phenotypes. This new concept may transcend ASD and apply to other neuropsychiatric disorders with a strong male bias (i.e., 2–4-times), such as intellectual disability, attention-deficit hyperactivity disorder, and schizophrenia (Abel et al., 2010; May et al., 2019).

In summary, our results indicate that the CHD8-S62X mutation in mice leads to age-differential sexual dimorphism in behavioral deficits.

## Data availability statement

The original contributions presented in the study are included in the article/Supplementary material, further inquiries can be directed to the corresponding author.

## Ethics statement

The animal study was reviewed and approved by Committee on Animal Research at KAIST.

## Author contributions

SL and HKw performed mouse behavioral experiments. HKa and EK wrote the manuscript. All authors contributed to the article and approved the submitted version.

## Funding

This work was supported by the IBS-R002-D1 (to EK).

## Conflict of interest

The authors declare that the research was conducted in the absence of any commercial or financial relationships that could be construed as a potential conflict of interest.

## Publisher’s note

All claims expressed in this article are solely those of the authors and do not necessarily represent those of their affiliated organizations, or those of the publisher, the editors and the reviewers. Any product that may be evaluated in this article, or claim that may be made by its manufacturer, is not guaranteed or endorsed by the publisher.

## Supplementary material

SUPPLEMENTARY FIGURE 1 Levels of *Chd8* mRNAs in *Chd8^+/S62X^* male and female mice. **(A)** Nucleotide sequence analysis of the CHD8-S62X mutation in *Chd8 ^+/S62X^* mice. The WT sequence is also indicated for comparison. Examples shown are from WT and *Chd8 ^S62X/S62X^* mice at the age of P0. Orange, WT nucleotide (TGA); blue, mutant nucleotide (TCA). **(B)** Quantification of *Chd8* mRNAs in male and female WT and *Chd8 ^+/S62X^* mice at the ages of P0, P25, and P80. Note that there are increases in *Chd8* mRNA levels in both male and female *Chd8 ^+/S62X^* mice at P25 and P80 but not at P0, which likely represent compensatory upregulation at the mRNA levels. (*n* = 3 mice [male-WT], 3 [male-HT/heterozygote], 3 [female-WT], and 3 [female-HT], ***p* < 0.05, ****p* < 0.001, two-way ANOVA with Holm-Sidak test).

SUPPLEMENTARY TABLE 1 Statistical details.
