# Supplementary figures and images for: Age-differential sexual dimorphism in CHD8-S62X-mutant mouse behaviors
Source: Front Mol Neurosci. 2022 Oct 25;15:1022306. doi: 10.3389/fnmol.2022.1022306 (PMC9641250; doi:10.3389/fnmol.2022.1022306)

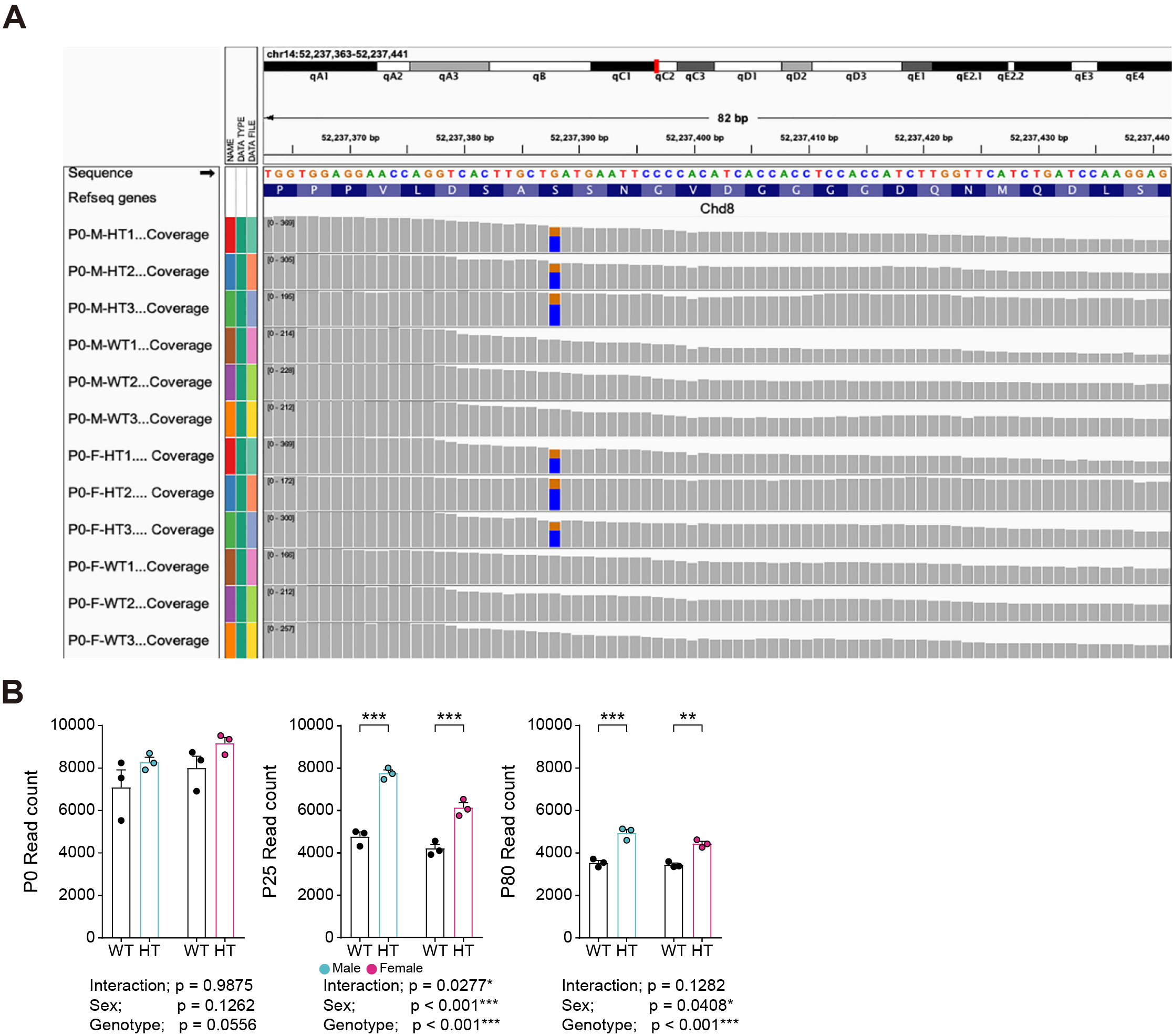

Supplement: SUPPLEMENTARY FIGURE 1 — Levels of Chd8 mRNAs in Chd8+/S62X male and female mice. (A) Nucleotide sequence analysis of the CHD8-S62X mutation in Chd8 +/S62X mice. The WT sequence is also indicated for comparison. Examples shown are from WT and Chd8 S62X/S62X mice at the age of P0. Orange, WT nucleotide (TGA); blue, mutant nucleotide (TCA). (B) Quantification of Chd8 mRNAs in male and female WT and Chd8 +/S62X mice at the ages of P0, P25, and P80. Note that there are increases in Chd8 mRNA levels in both male and female Chd8 +/S62X mice at P25 and P80 but not at P0, which likely represent compensatory upregulation at the mRNA levels. (n = 3 mice [male-WT], 3 [male-HT/heterozygote], 3 [female-WT], and 3 [female-HT], **p < 0.05, ***p < 0.001, two-way ANOVA with Holm-Sidak test). [file Image_1.TIF]
